# Shared decision making: audiology student perspectives

**DOI:** 10.3389/fresc.2023.1254836

**Published:** 2023-11-14

**Authors:** S. Hussain, C. Wilkes, N. Dhanda

**Affiliations:** ^1^ Department of Audiology, School of Life and Health Sciences, Aston University, Birmingham, United Kingdom; ^2^ College of Medical and Dental Sciences, Institute of Applied Health Research, University of Birmingham, Birmingham, United Kingdom

**Keywords:** shared decision making, students, healthcare, audiology, education, training, person centred care

## Abstract

**Introduction:**

Shared decision making is a concept in healthcare that actively involves patients in the management of their condition. The process of shared decision making is taught in clinical training programmes, including Audiology, where there are several options for the management of hearing loss. This study sought to explore the perception of Healthcare Science (Audiology) student views on shared decision making.

**Methods:**

Twelve students across all years of the BSc Healthcare Science degree took part in three semi-structured focus groups. Four students were work-based learners, and eight students were enrolled on the standard pathway. Data were analysed using Thematic Analysis.

**Results:**

Students’ definition and understanding of shared decision making was influenced by three key factors that were based on using a range of resources, implementation of a decision aid, and recognising Ida Institute as a pinnacle of shared decision making. Students also identified their roles as the future of healthcare workforce and the importance of disseminating best practice.

**Conclusion:**

Shared decision making is valued by students in their roles as healthcare trainees. This study data will enhance teaching practices for healthcare science students in audiology training. Future research involving patient views in clinical training is vital.

## Introduction

### Person-centred care in healthcare

Person-centred care (PCC) is an approach to healthcare that prioritises the individual needs, preferences, and values of patients who are seeking health services (1). Within Audiology, PCC recognises that each person is unique and that their experiences, communication needs, and goals should be at the centre of their audiological care (2). Some of the key principles and elements of person-centred care in Audiology include holistic assessment, active listening, individualised treatment plans, education, and shared decision making (SDM) (3). Whilst most clinicians agree with the importance of PCC, consistent implementation is a challenge (4). Some elements of PCC may be used more frequently than others, largely dependent on time available and clinician experience (5). Nevertheless, the emphasis of PCC within healthcare has encouraged clinicians to use SDM amongst other aspects to improve their delivery and outcomes of hearing healthcare (6).

### Shared-decision making as a component of person-centred care

Shared decision making (SDM) is a collaborative approach in healthcare that involves active participation of both patients and healthcare providers in making informed decisions regarding management options (7). In audiology, where decisions regarding hearing healthcare have a significant impact on individuals’ quality of life, implementing PCC and more specifically, SDM, is crucial because it places the patient at the centre of their care (8). SDM acknowledges the unique preferences, values, and goals of individual patients, ensuring their active involvement in the decision-making process (9). In audiology, patients vary in their hearing needs, lifestyle, communication preferences, and financial constraints (10). SDM and other components of PCC help clinicians understand these factors and tailor treatment plans, accordingly, with the aim of improved patient satisfaction and better treatment outcomes.

Audiological decisions often involve complex choices related to hearing aids, cochlear implants, communication strategies, and rehabilitation options (10). Through SDM, clinicians can educate patients about these choices, providing them with accurate and understandable information about the benefits, risks, and alternatives (11). Patients who are well-informed are more likely to actively engage in their care, have realistic expectations, and make decisions that align with their personal preferences (12).

### Benefits and barriers of shared decision making

The many benefits of SDM seemingly outweigh the challenges and barriers though. When patients are actively involved in decision making, they are more likely to adhere to the co-constructed management plan (13). SDM allows patients to express their concerns, address potential barriers, and actively participate in setting achievable goals (8). By understanding their management options and having their preferences considered, patients feel empowered and invested in their hearing healthcare journey (11). What's more, SDM fosters effective communication between clinicians and patients (14). It encourages open dialogue, active listening, and shared understanding. Through SDM, clinicians can gain insights into patients’ values, expectations, and concerns, which can be integrated into the decision-making process. This patient-clinician partnership builds trust, strengthens the therapeutic relationship, and ensures that decisions are based on accurate and shared information (15). Individuals may have different goals and priorities when it comes to their care, and SDM can help to ensure that these are taken into consideration. For example, some patients may prioritise improving their hearing in noisy environments, while others may prioritise the ability to hear music or speech clearly. By involving patients in the decision-making process, healthcare professionals can better understand these preferences and tailor treatment plans accordingly (11).

### What is involved in teaching SDM?

For SDM to be implemented well in audiology clinical practice, trainee clinicians must be taught the appropriate skills to deliver it. Clinical skills play a vital role in audiology education as they allow students to develop the necessary competencies to provide effective patient care (16). These skills encompass various aspects, such as conducting assessments, diagnosing hearing disorders, fitting hearing aids, counselling patients, and conducting rehabilitation programs.

Audiology programs typically employ a combination of didactic (classroom-based) and clinical (practical) teaching methods to help students acquire clinical skills (16). Didactic components include lectures, seminars, and workshops to provide theoretical knowledge, while clinical components involve hands-on training in audiology clinics or simulated environments (17). To enhance students’ clinical skills, audiology programs often require students to complete supervised clinical placements. These placements allow students to apply their knowledge in real-world settings under the guidance of experienced audiologists. During these placements, students may interact with patients, perform diagnostic tests, assist in hearing aid fittings, and observe various audiological procedures (18). Most importantly, the expectation is that students encompass SDM into their clinical training (19).

PCC as a whole requires specific skills, such as effective communication, information sharing, eliciting patient preferences, and facilitating deliberation (20). Teaching these skills and providing trainees with opportunities to practice and receive feedback can be challenging, as it requires dedicated time and resources for training and mentorship (7). Moreover, teaching SDM within the constraints of a busy clinical environment can be difficult (21). Balancing the need to provide comprehensive care within limited appointment durations may leave limited time for in-depth discussions and SDM. Incorporating SDM training into already packed curricula can also pose challenges.

To support students’ learning and understanding of SDM, a variety of teaching methods are used (22). Simulation-based training has gained prominence in audiology education (23). Simulated environments, including virtual patients or computer-based simulations, provide a safe and controlled space for students to practice clinical skills. This approach allows students to develop competency and confidence before working with actual patients. A competency-based approach focuses on developing specific skills and abilities required for audiology practice (24). Competency frameworks outline the essential skills, knowledge, and behaviours expected of audiology professionals (25). These frameworks guide curriculum development and assessment strategies to ensure students meet the necessary standards in clinical practice. Reflective practice is often integrated into audiology education to enhance students’ self-assessment and critical thinking skills. Through reflective exercises, such as journaling or group discussions, students can analyse their clinical experiences, identify strengths and areas for improvement, and develop strategies to enhance their clinical skills (26).

### Decision aids

Decision aids are a common tool used to support SDM and PCC in healthcare settings (27). Using a hearing decision aid in audiology is of great importance as it plays a crucial role in facilitating SDM, improving patient satisfaction, and enhancing clinical outcomes (28). A hearing decision aid is a tool or resource that helps individuals with hearing loss and their healthcare providers make informed decisions about their hearing healthcare (11). A hearing decision aid empowers patients by providing them with relevant information about their hearing loss, management options, and potential outcomes (28). It serves as an educational tool that helps patients better understand their condition, the available interventions, and the associated benefits and risks. With this information, patients can actively participate in the decision-making process, ask informed questions, and express their preferences and concerns.

A hearing decision aid promotes SDM between patients and healthcare professionals. It facilitates open and collaborative discussions, allowing patients to express their needs, values, and goals (29). Healthcare professionals can then provide tailored recommendations and interventions based on the individual patient's preferences and circumstances. This SDM approach not only improves patient satisfaction but also enhances treatment adherence and outcomes (8). When patients are actively involved in decision making, they are more likely to be committed to their management plan, leading to better overall hearing healthcare management (10).

At our university, we teach students to use an audiology SDM decision tool focussed on hearing loss management options from their first year of study (10). There are a few variations of the decision tool that we use. We have chosen an evidence-based hearing loss management decision tool that has relevance within the UK. The decision aid is essentially a one-page grid that lists the different management options for hearing loss alongside a description, the advantages and disadvantages, and how the option can be accessed. There are students following the traditional undergraduate route, and students undertaking a Degree Apprenticeship, where much of their learning and training is ‘on-the-job’ and much of their university attendance is for theoretical learning. There is a need to understand students’ perspectives of SDM and how this translates to the clinical workplace. To our knowledge, there is little to no literature in this area. A study exploring the preference of patient-centredness (akin to SDM) in undergraduate Audiology students (30) found that patient-centeredness is influenced by audiology education. Within the first year of undergraduate study, students establish a strong preference for patient-centeredness, which remains steady throughout the course of four years. However, it is not clear what happens during clinical placement or beyond graduation. There is a significant need to understand the student experience and their understanding of SDM.

## Methods

### Approach

Three focus groups were conducted, lasting one hour each. All participants were allocated to a focus group according to their academic year group. The focus groups were facilitated by SH and CW, who are Senior Teaching Fellows that are responsible for teaching communication and person-centred care in Audiology Programmes. They have experience of working as facilitators and have been involved in the implementation of Patient and Public Involvement strategies for Audiology. This meant they were able to empower the participants to engage in the focus groups. They also ensured that all the participants contributed evenly, by asking the same questions to all participants and ensuring all participants had an equal voice (as outlined by the ground rules before the focus group commenced).

We adopted a qualitative methodology, informed by Grounded Theory (31), to understand students’ perspectives of SDM. This methodology utilises a constant comparison approach across different student cohorts using systematic and iterative processes. Focus groups were used to understand the student experience and give voice to their learning. We used open coding, constant comparison and axial coding to develop the Grounded Theory model to identify the contributing factors and central phenomenon.

We ensured that we had student representation from across all undergraduate year groups, including work-based learners. We felt that it was imperative that all students felt confident to speak honestly and freely. To enable this, we held the focus groups in rooms that the students were used to and felt comfortable in. The students had experience of communicating with each other and were known to each other as they are taught together throughout the Audiology Degree. The students had experience of communicating with the facilitators as they were both key members of the teaching team and taught the students on a regular basis. A semi-structured interview schedule was used within each of the focus groups. Ethics for this study was sought and approved by The College of Health and Life Sciences Research Ethics Committee at Aston University (REC ID: HLS21032).

### Reflexivity

Researchers who conducted the focus groups included a qualified Lay Counsellor (CW) and Lead Clinicians (CW/SH/ND) responsible for the teaching and research of communication skills to the students. The researchers have an established interest in Person Centred Care and were founding members of the international Person-Centred Care Network for the Ida Institute. It was their passion of person-centred care and Shared Decision making that led to implementation of this research into students understanding of SDM. It was this experience that allowed staff to identify on group dynamics, allowing students to have the confidence to speak to known staff on this topic. It is important to note that all researchers have a clinical background in audiology. Therefore, reflexivity was key in acknowledging the role that each clinician played were considered during data analysis (32).

### Data gathering

Focus group 1 consisted of work-based learners. Focus group 2 consisted of final Year BSc Students and focus group 3 consisted of first- and second-year BSc students combined. Final year students and work-based learners generally have the confidence and skill set to talk widely in a group setting. To enable the younger year groups to feel supported and enabled to do this, the decision was taken to group first and second years together for their focus group.

Students were recruited through opportunity sampling. An advert for the study was placed on the Virtual Learning Environment, Blackboard, through a shared handbook space. Students were required to email the staff members (in this study) if interested in participating in the focus groups. A stratified sampling method was used to ensure adequate representation from each year group. To ensure the sample was representative we needed to recruit a minimum of four work-based leaners (apprentices), four final year students and four first and second years combined. This accounted for 15% of the total BSc cohort 2022-2023. This objective was achieved.

It was essential that the focus groups were accessible to all interested students, to enable this the decision was made to host the focus groups with the option of being face to face or online through Microsoft Teams as this was a platform taught to students. The only focus group that resulted in a mix of online and face to face participants was focus group one with the work-based learners. This was not challenging to manage as work-based learners are experienced in working with professionals and patients using hybrid technology. The facilitators and participants were able to follow the ground rules set to ensure everybody engaged in the discussion and had an equal voice. Table 1 provides a list of question prompts that were used in the focus groups. The questions were formulated and chosen based on informal discussions with Audiology students across the program in the Clinical Skills laboratory setting. The decision was made to make reference to decisions aids as part of the SDM concept as this was something tangible that the students could easily grasp and talk about.

**Table 1:** Focus groups question prompts.

Question 1	What do you understand by the term shared decision making?
Question 2	Have you observed a clinician with a patient when person-centred care and shared decision making were followed/implemented?
Question 3	How do you have those discussions with patients? What tools are available to help you have the discussion about what options are available?
Question 4	What value do you think decision aids have in clinical settings? Thinking specifically of the Hearing loss: hearing technologies options decision aid.
Question 5	What factors do you think helped the use of a decision aid and other resources? What factors do you think hindered?
Question 6	How do you view your role as a student in embedding shared decision making and using decision aids?
Question 7	What would help your confidence in using decision aids?
Question 8	Is there anything that would support you in your clinical skills training in using decision aids?

All audio recordings were sent for transcription through an approved university supplier. All transcripts were anonymised and checked for accuracy. Manual analysis was utilised to allow the researchers to re-familiarise themselves with the discussions held in the focus groups. All student data was kept anonymous and all focus groups were conducted in English as participants were enrolled on a UK degree programme.

Each focus group transcript was coded independently, and the codes were compared across transcripts. Researchers SH and CW conducted the initial coding, followed by blind-coding with researcher ND to guarantee uniformity across the codes through the process of triangulation. The open coding retained a broad view of all theoretical directions and prioritised the preservation of the participants’ language (33). Notes and memos were also referred to here. The researchers reduced these codes into bigger categories (axial coding) by investigating their features and dimensions in relation to the central phenomenon. The relationships between these categories were investigated in order to create a model of contributing factors towards student understanding of SDM.

## Results

There were twelve participants in total. All participants were enrolled on the BSc Healthcare Science Degree at Aston University, Birmingham. There were three focus groups consisting of four work-based learner students on the apprenticeship training route and eight students on the standard undergraduate route. The work-based learners were employed in National Health Service departments across the UK (Southeast, West Midlands and South of England). The four Year 3 standard route students were interviewed before their final year clinical placements. Eleven out of the twelve participants were female.

**Figure 1:**
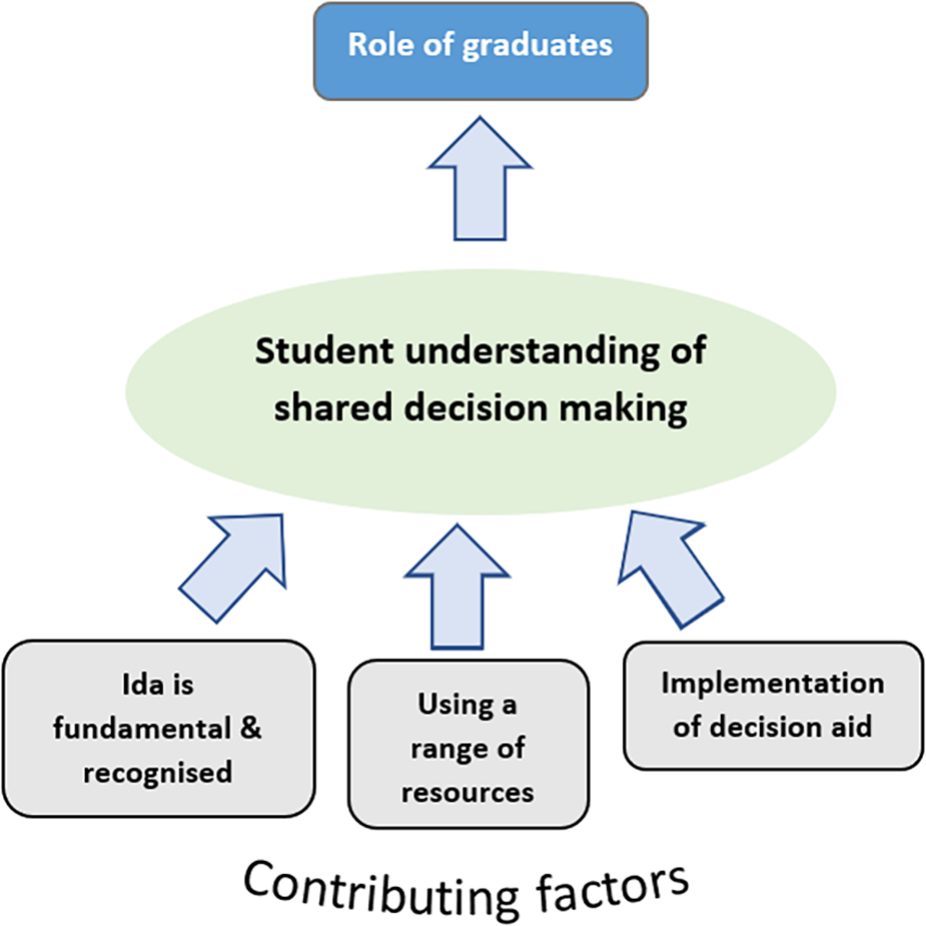
Model for student understanding of shared decision making.

The key themes that participants discussed in the focus groups are discussed below. The relationship between these themes is depicted in a theoretical model illustrated in Figure 1.

### Findings

The core themes were organised into a model, informed by Grounded Theory. The central phenomenon was student understanding of SDM. Specifically, how students conceptualise and define the or process of SDM. This described the main features of students’ perceptions of SDM and the corresponding influencing considerations considered, which this paper terms as ‘contributing factors’.

To maintain anonymity, all quotes provided do not include names, only the participant group.

### Student understanding of SDM

The central phenomenon of the findings was student understanding of SDM. Student understanding of SDM was a process of internalisation of the concepts, theoretical teaching and patient experience surrounding SDM experiences, plus opportunities to enact SDM. This was characterised by their definitions that included the role of the clinician and the role of a patient. Informed choice and/or decision was highlighted by the students as a key feature in their definitions.

“My understanding of shared decision making is especially in the context of a clinic, we as a clinician are dictated by what the patient needs to do, it's a shared journey, it's their care, they have a say in their own healthcare so we need to give them enough information to make an informed decision and guide them in that direction.” (Work-based learner).

“It's about the clinician being able to share their knowledge and come to a decision with the patient, based on patient needs and wants, putting the pieces of the puzzle together and coming up with a plan going through the pros and cons of the options so they can make an informed decision.” (Work-based learner).

The types of definitions provided for SDM were consistent across all year groups despite students having varied exposure to patient clinics and patient interactions. Students further noted the weighing up of pros/cons to decision options. Interestingly, students highlighted the passivity of their own roles, with the outcome decision making, and work, being the responsibility of the patient.

“We have to give all the audiological knowledge that we know but ultimately it's still their decision, we’re there to answer questions and provide options.” (Year 3)

“We go to clinic or to the hospital for placement, it will be very different because they’re real people and when I went to work last year, I could see that patients really needed help, “what do I do?” (Year1/2)

### Ida is fundamental and recognised

This theme is a contributing factor to the definition of SDM identified above. The Ida institute is an independent non-profit organisation specialising in person-centred care (34). The Ida Institute resources used in clinical teaching were signposted by several students as an influential factor in their audiology training.

“Ida tools, we did a bit about them in our modules” (Year 2)

The Ida institute were also referred to as the authority figure on the position and practice of shared decision making.

“Ida gave the talk on patient centred care and that's why we associate Ida with shared decision making.” (Year 3)

Clinical tutors often discuss Ida with high esteem, because of their ethos around person-centred care. The positivity and respect for Ida trickles down to students and appears to cement an associated trust with Ida and a likens its work and mission to SDM.

### Using a range of resources

The second contributing factor to the definition of SDM was the process that students foster when choosing which resources to use in their clinical settings. The array of resources available provides students and clinicians alike with a degree of autonomy during appointments. These included resources from different charities and institutions. Students discussed their preferences for choosing literature based on the author of resources (e.g., trusted or well-known charities and organisations). Students also recognised the importance of patient-friendly language in the materials provided to patients.

“There's so many different types of hearing aid, it's like learning a new language, so much jargon and abbreviations that patients have to learn, having these resources available to them can help them understand things better.” (Year 3).

Participants also referred to the value of significant others/partners of patients. This was identified as another resource that students utilised in their training.

“…letting them know they can change their decision and opt for something else if they think it's not working, also with the friends and family.” (Work-based learners)

“They're so easy to read, they can show family members, it's not too wordy and the jargon is simple.” (Year 3)

### Implementation of decision aid

The third and final influencing factor to the definition of SDM was the use of decision aids, notably/specifically the Hearing Aid Technology Options Grid (12). This is a tool used in clinical context with patients and taught to students. Students reported on their utilisation of the options grid in their training or observed in real life settings. These were highlighted as being valuable in facilitating an appointment.

“They're very important, because most patients don't know exactly what to do straightaway, having a decision aid that's very clear, easy to follow and easy to explain and it's something they can take away and read.” (Year 3)

“A decision aid adds great structure to the appointment, it's a nice summary and it can make the patient feel like they’ve been listened to because their options are put together, tailored to them and so they feel included.” (Work-based learners)

Students recognised the decision aid is an iterative process and can be referred to in subsequent appointments multiple times.

“As a clinician, we need to think about when it is appropriate to use it, the patients could come back for a repair and you might show them the options again.” (Year 3)

“You can show them the decision aid in another appointment, maybe when it’s not working for them, you can tell them about the other options.” (Year 3)

### The role of graduates

The final theme pertains to the responsibility students faced in their current training positions. Students seemed to internalise the obligation they face in their implementation and dissemination of patient centred practices.

“Us as the new generation have a bit of responsibility because we’re doing this and it's not our place to implement it, but we can get it out there and known that this is a better way to work.” (Work-based learners)

“I think we should be using these decision tools more; we need to educate audiology departments and eventually, it will be something that they have to implement to include the patient and make it more patient centred.” (Work-based learners)

Some students faced challenges when they were in clinical environments where SDM was not frequently adopted or used at all.

“I do feel there’s been a disconnect between what we learn and what we do at work, departments have their own guidelines and you sometimes may learn something at uni and you're trying your best to do everything right and not pick up any bad habits, but when you get to work, it’s still safe but they do it differently so you're having to choose between doing it how you've been taught and keep up that practice.” (Work-based learners).

## Discussion

### Importance of SDM

Students highlighted their influencing factors in how they understand and practice SDM based upon their clinical training with patients and simulated patients. This an important factor in clinical training with an emphasis on patient interaction required in healthcare education (35, 36). SDM is of paramount importance in audiology as it enhances patient-centred care, facilitates patient education, improves treatment adherence, strengthens communication, and upholds ethical standards.

Whilst many healthcare courses teach SDM principles (37, 38), to the knowledge of the authors, this is the first study to investigate audiology students specifically. We have modelled the process by which students define SDM and practice this. This is important because audiology is one of the few professions in healthcare that is involved in the diagnosis and rehabilitative aspects of patient care. Emphasising SDM in audiology practices can positively impact the lives of individuals with hearing loss, ensuring that their treatment choices align with their values, preferences, and goals. Students in this study were cognisant of their roles in the future healthcare workforce and their contribution to service improvement and patient care.

### Barriers to SDM

Students in this study highlighted barriers to implementing SDM practices. For some clinicians, the challenges of administering SDM in clinical practice can outweigh the desire to conduct it (39). This is often borne from the perceived time constraints and limited appointment durations, particularly within publicly funded healthcare models such as the National Health System in England (40). Engaging in SDM requires allocating sufficient time for discussion, exploring options, and addressing patient concerns. However, time limitations can hinder the process, leading to rushed decisions or incomplete discussions (15). Having said that, evidence suggests that if clinicians use their appointment durations differently, spending more time on history-taking and SDM, an accurate diagnosis can still be sought because of the rich information gathered from listening to the patient (2).

Teaching SDM to trainee clinicians can present several challenges (41). Trainee clinicians may have limited exposure to SDM principles and practices during their education. Students in this study were at various stages of their programme where Year 3 students undertake a several month placements in a clinical setting with patients. This study was conducted prior to the Year 3 students beginning their placement. Trainee clinicians often learn by observing and emulating their supervisors and experienced clinicians. If the practicing clinicians themselves do not consistently demonstrate and prioritise SDM, it can create a mismatch between what is taught and what is observed, making it harder for trainees to internalise SDM principles (38).

### Student cohorts

The work-based learner participants reported on the variety of approaches to SDM exhibited by qualified professionals in their clinical settings. This is mirrored within traditional medical education has historically emphasised a more paternalistic model of decision making (42), which can make it challenging to introduce the concept of SDM as a core skill. Shifting from a traditional, clinician-driven approach to a collaborative SDM model requires a significant shift in mindset and practice. There is a hidden curriculum sometimes present within clinical supervisor-supervisee relationships whereby students are expected to purposefully mimic the practice of their clinical supervisors (and the wider team), rather than utilise their theoretical learning to develop their own way of working (43). When this occurs, there are tensions and dilemmas about implantation of best practice, and distinction between evidence-based practice vs. practice-based evidence (44). Furthermore, Some trainees may be resistant to change or may struggle to adopt a more patient-centred approach due to ingrained beliefs, hierarchical structures, or concerns about relinquishing control over decision making (20). Our students all reported on their drive to propagate SDM in their future clinical roles. This was balances however This is particularly evident in some students’ experience of learner conflict or tensions between their understanding of ‘best practice’ at university compared to the realities within clinical practice (45).

### Use of tools to support SDM

Students associated implementing SDM with the use of tools such as decision aids and other literature. This varied amongst the cohorts in line with the level of experience the students were exposed to. In audiology, there is often complex information related to various hearing aid technologies, performance metrics, and other management options (46). Students recognised this information can be overwhelming and challenging for patients to comprehend. However, students reported on how hearing decision aid simplifies and presents this information in a user-friendly and accessible manner. Students also reported on how it helped them to structure an appointment. This is supported by literature suggesting healthcare professionals also benefit from using a hearing decision aid (14). It supports their communication with patients, allowing for efficient and effective counselling sessions. By utilising a decision aid, healthcare providers can ensure that patients receive consistent and accurate information, reducing the potential for misunderstandings and miscommunication (47). Furthermore, a hearing decision aid can serve as a documentation tool, capturing patient preferences, decisions, and shared goals. This documentation can enhance continuity of care and provide a reference for future discussions and treatment adjustments (48).

Students also reported on the importance of supporting patients with materials to take home, to share with families or by providing extra resources where English may not be the patient's first language. The availability of resources, such as educational materials, decision aids, and information about different treatment options, may vary across audiology clinics. Limited access to these resources can hinder the implementation of SDM, as patients may not have access to comprehensive and unbiased information to support their decision-making process (49). Socioeconomic and cultural factors can contribute to health disparities and impact SDM (47). Trainees encounter patients from diverse backgrounds and with varying levels of health literacy and cultural beliefs (50). Patients from marginalised communities may face additional barriers, such as lack of access to healthcare, health literacy issues, and distrust in the healthcare system (51). Tailoring SDM approaches to meet the needs of diverse populations and addressing potential communication and cultural barriers can pose challenges in training. Addressing these disparities is crucial to ensure equitable SDM in audiology clinical practice.

## Limitations

This study was limited to one academic institution based in the United Kingdom. This is of relevance due to the National Health Service (NHS) which is the main training and employing organisation of student and graduate audiologists. Therefore, whilst the authors feel the findings from this study are translational to other countries, it is important to be aware of the nuances in different healthcare systems and its applicability.

Furthermore, some student cohorts had not yet experienced real clinical settings in their training at the time this study was conducted. The authors set out to include all year groups in this study to ascertain the perspectives from different stages of their education and training. The findings support that the appreciation for SDM arose in Year 1 of the course and similar definitions were provided across all the year groups, irrespective of length of clinical exposure and training.

The authors in this study who facilitated the focus groups are known to the students. This was felt appropriate due to the topic investigated in this study. However, it is worth noting and exploring if unfamiliar staff facilitators may have elicited alternative responses. The authors discussed their involvement in the reflexivity section and do not believe their involvement deterred students from responding. It is also worth noting that students who participated in the study are keen to explore SDM and enhance their own clinical development. Therefore, future research needs to target a range of students. This is the first study of its kind and thus these practical considerations have implications for future research.

## Conclusion

In conclusion, this study highlights that Audiology students recognise the importance of SDM and PCC, however they rely on tangible resources in the tailored application of these concepts. Students recognise the importance of SDM in their approach to healthcare as this can provide significant benefits to patients. These audiology students reported SDM can help to ensure that patients are actively involved in their care, make more informed decisions about their hearing healthcare, and have their values and preferences considered. By implementing SDM early in audiology teaching, students and graduates can improve the overall quality of care provided to patients and foster a sense of trust and collaboration between patients and themselves as healthcare professionals. By supporting students in their clinical training, students can empower their patients, improve treatment outcomes, and contribute to a more patient-centred system.

### Future directions

This study highlights the influencing factors in defining SDM in undergraduate students. Future research should investigate the views of postgraduate students as well as other work-based learning programmes across the UK audiology landscape. Patient interactions and clinical supervisors are an integral aspect of clinical training and should also be invited to participate in enhancing audiology education and subsequently, patient care. The findings of this research will support higher education teaching staff in clinical teaching of SDM. There will also be greater insight into clinical supervision and understanding of SDM.

## Data Availability

The original contributions presented in the study are included in the article/Supplementary Material, further inquiries can be directed to the corresponding author.
